# Seeding Chiral Ensembles of Prolinated Porphyrin Derivatives on Glass Surface: Simple and Rapid Access to Chiral Porphyrin Films

**DOI:** 10.3389/fchem.2021.804893

**Published:** 2022-01-31

**Authors:** Gabriele Magna, Tanja Traini, Mario Luigi Naitana, Gianlorenzo Bussetti, Fabio Domenici, Gaio Paradossi, Mariano Venanzi, Corrado Di Natale, Roberto Paolesse, Donato Monti, Manuela Stefanelli

**Affiliations:** ^1^ Department of Chemical Science and Technologies, University of Rome Tor Vergata, Roma, Italy; ^2^ Department of Physics, Politecnico di Milano, Milan, Italy; ^3^ Department of Electronic Engineering, University of Rome Tor Vergata, Roma, Italy; ^4^ Department of Chemistry, Università La Sapienza, Roma, Italy

**Keywords:** porphyrins, drop-casting, solvent effect, glass surface, self-aggregation, dynamic light scattering, supramolecular chirality

## Abstract

An easy and fast method to achieve chiral porphyrin films on glass is herein reported. The on-surface formation of organized supramolecular architectures with distinctive and remarkable chiroptical features strictly depends on the macrocycles used, the solvent chosen for the casting deposition, and most importantly, on the roughness of the glass slide. Dynamic light scattering studies performed on 10^−4^–10^−6^ M porphyrin solutions revealed the presence of small porphyrin aggregates, whose size and number increase depending on the initial concentration. Once transferred on surface, these protoaggregates act as nucleation seeds for the following, self-assembling into larger structures upon solvent evaporation, with a process driven by a fine balance between intermolecular and molecule–substrate interactions. The described method represents a straightforward way to fabricate porphyrin-based chiral surfaces onto a transparent and economic substrate in few minutes. The results obtained can be particularly promising for the development of sensors based on stereoselective optical active films, targeting the detection of chiral analytes of practical relevance, such as the so-called emerging pollutants released in the environment from agrochemical, food, and pharmaceutical manufacturing.

## Introduction

**SCHEME 1:**
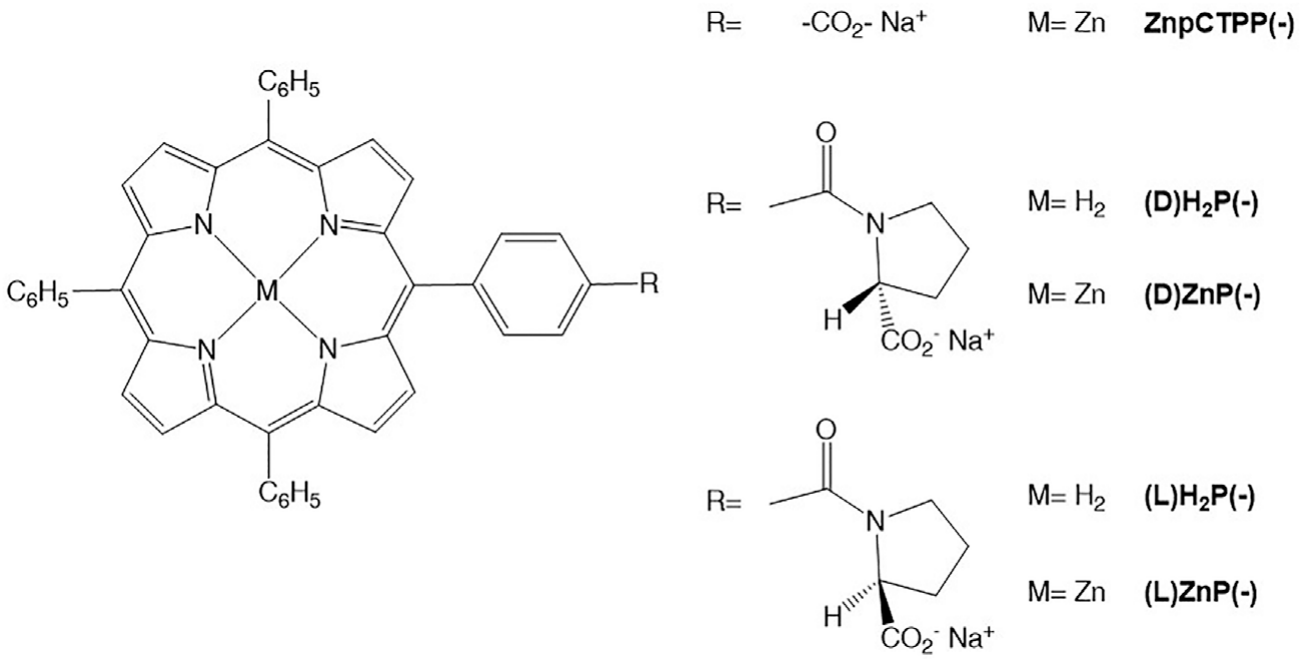
Porphyrin derivatives used in these studies.

The understanding of chirality at solid surfaces is of ever-growing importance, and an increasing number of studies have been reported over the years to shed light on the fundamentals and practices in achieving chiral surfaces with definite enantioselectivity (Gellman, 2010; Zaera, 2017; Albano et al., 2020; Xu et al., 2021). As a matter of fact, chirality can be observed or provided to achiral surfaces taking advantage of several options spanning from the addition of chiral modifiers (Zaera, 2008), the adsorbing of chiral molecules at ultra-high vacuum (Costa et al., 2015), or from liquid phase (Huang et al., 2003), or even by self-assembly of achiral molecules that form chiral domains thanks to the surface dimensional constraints (Tao and Bernasek, 2005). Specifically for the transfer of chiral molecules from solution to surface, the final chiroptical features are strictly dependent on a combination of different forces controlling the supramolecular organization in solution as well as the interaction/adsorption with the specific substrate. Over the course of our research, we found that various amphiphilic porphyrin derivatives bearing a stereogenic information on the peripheral molecular positions form chiral mesoscopic structures of tunable size in different hydro–organic media, depending on the interplaying of structural motifs and solvent bulk properties (i.e., type and relative ratio of the hydro–organic mixture used, ionic strength, and monomer concentration) (Monti et al., 2010; Caroleo et al., 2019; Stefanelli et al., 2020a). Within this set of tetrapyrroles, cationic derivatives functionalized with a peripheral (L)-proline moiety have been also exploited for the preparation of chiral films to be applied for sensor development. Two strategies have been basically reported: 1) the steering of the self-aggregation of chiral porphyrin monomers in ethanol/water mixture of proper composition to give in 1–2 days chiral assemblies that spontaneously layer on glass substrate dipped into the solution (Monti et al., 2011); 2) the use of Langmuir–Blodgett technique to assemble the porphyrin molecules onto a glass surface in chiral multilayers through air/water interfacial organization (Colozza et al., 2019). Films based on the anionic counterpart (L)ZnP(-) (Scheme 1) anchored to ZnO nanoparticles have been also chirally organized on quartz microbalance surfaces and used for the stereoselective recognition of limonene enantiomer vapors (M. Stefanelli et al., 2019). The need of realizing cost-effective and easy to handle sensor platforms prompted us to access the chiral films on common solid supports by simple operation of all the stages, starting with material preparation to film deposition. Additionally, the achievement of a convenient signal for the detection is highly desirable. With this aim in mind, we have carried out the studies herein reported, concerning the development of a straightforward method to fabricate optically active films on glass by drop-casting of (L) or (D)-prolinated porphyrin building blocks dissolved in suitable organic solvents (Scheme 1). The choice of glass as substrate for film deposition is mainly due to its optical transparency that makes the layer chirality assessment possible by circular dichroism (CD) spectroscopy on one side, along with the detection of the eventual interaction with a target analyte by means of an easily recordable optical signal change (i.e., absorption, fluorescence, and CD spectral variations upon analyte detection). We can anticipate that the chiroptical properties of the produced films are deeply influenced by the porphyrin molecular structure (i.e., the configuration of the appended proline and the presence or not of a coordinated metal ion into the core), the solvent used for the deposition, and last but not the least, the roughness of the glass substrate. These findings highlight the complex interplaying of supramolecular interactions occurring when chirality is transferred from the solution to the solid state. The easiness and the versatility of the described protocol make it a viable approach in fabricating chiral films based on porphyrins for stereoselective sensor applications.

## Results and Discussion

### Evaluation of the Solvent Effect on the Chirality of the Film

The challenging stage for the drop-casting production of chiral solid films is surely the identification of the proper solvent to use for the stock solution. As reported in the literature, the nature of the solvent used is extremely relevant to define the structures that the layers adopt and the overall chirality (Iavicoli et al., 2009; Chen et al., 2019), since both derive from a combination of the interaction strength of the molecules with the surface and their solubility (Zaera 2009; Mali and De Feyter, 2013). Moreover, solvent parameters like viscosity, surface tension, and vapor pressure strictly impact the film adhesion to the substrate and the evaporation rate, producing layers featuring different structural motifs and homogeneity (Resedean et al., 2020).

We carried out our investigations on the Zn porphyrin enantiomers [(D)- and (L)ZnP(-), Scheme 1], whose aggregation behavior in hydroalcoholic solution was recently studied in depth (Stefanelli et al., 2020a; Savioli et al., 2020). We have selected three solvents of different polarity, coordination abilities, and adhesion properties in which the porphyrin derivatives are soluble, namely, ethanol (EtOH), tetrahydrofuran (THF), and toluene. Films were deposited by drop-casting 10 μl from 10^−4^ M stock solutions of each porphyrin derivative on glass slides, followed by solvent evaporation (see the *Experimental* section for the detailed procedures). The layered materials were optically characterized by UV-vis and CD spectroscopies. For all the films tested, UV-vis spectra showed broad Soret bands, with peaks lying in the 435–440 nm range, red shifted if compared with the corresponding porphyrin solution (420–424 nm range), indicating that macrocycles are aggregated in J-type structures at solid state (see Supplementary Figure S1).

The assessment of the chiroptical features of the solid films have been carried out by CD spectroscopy and evidenced a remarkable influence of the solvent on the chirality at solid state.

**FIGURE 1:**
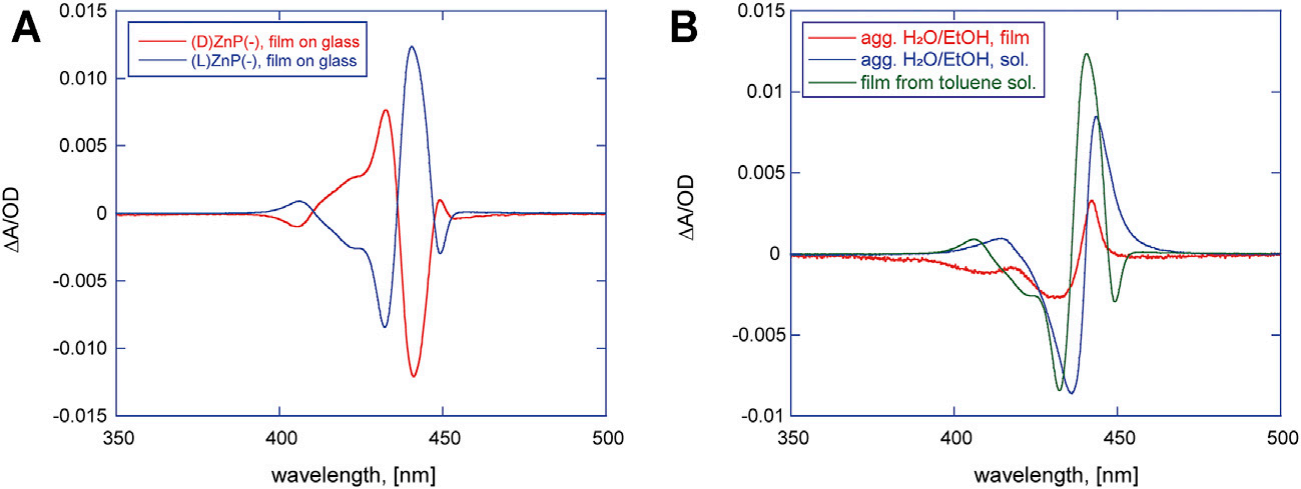
**(A)** Circular dichroism (CD) spectra of (D)- (red trace) and (L)ZnP(-) (blue trace) casted films on glass from 10^−4^ M toluene solutions. **(B)** (L)ZnP(-) chiral aggregates obtained in different conditions: aggregates grown from EtOH/H_2_O (25:75, v/v) at 5 μM concentration (blue trace) and the corresponding film on glass (red trace); chiral films on glass from 10^−4^ M toluene solution (green trace).

As far as the films of (D)- or (L)ZnP(-) from THF and EtOH are concerned, negligible dichroic bands, barely observable in the 350- to 500-nm spectral range (data not shown) have been recorded, indicating an ineffective reading out of the chiral information during the self-assembly process occurring onto the glass surface in these solvents. Completely different results were obtained for the drop-casting of toluene solutions, which conversely produced layers with extensive supramolecular chirality. As shown in Figure 1A, the CD spectrum for films of (D)ZnP(-) is characterized by two sets of excitonically coupled bands at about 441, 433, and 407 nm, indicating electronic coupling between layered macrocycles that arranged in a (+) clockwise (CW) mutual conformation, as excitonic theory states (Berova et al., 2000) (Figure 1A, red trace). For the other enantiomer, a specular CD profile is obtained, featuring three bisignate negative bands with the same crossover points found for the (D) counterpart**,** pointing out a (−) anticlockwise (ACW) mutual orientation of porphyrin macrocycles (Figure 1A, blue trace). CD readouts (theta, expressed in mdeg), have been converted in ∆A/OD, where OD is the optical density of the sample and ∆A the difference measured in the sample absorbance between the left and right polarization of light. For this normalization purpose, both CD and optical density of films have been evaluated by performing absorbance measurements with Jasco J-1500 without moving the glass slide in order to measure these two quantities on the exact same spot. Remarkably, we found out that the normalized CD values are unexpectedly reproducible over different areas of the spot, even more so by considering that simple drop-casting method for the coating is used. This robustness results in an almost flawless specularity of CD signals obtained for films produced by toluene solutions of the two enantiomers of ZnP(-) (see Figure 1A). These spectral features were obtained independently on the batch used for deposition with the same sign and intensities, to prove a good reproducibility of the deposition method. All these findings combined definitely pointed out that the final chirality of the films is driven by the specific peripheral stereochemical information of the single unit and transferred from molecular to aggregate level upon self-assembly process.

The dependence of the chiroptical features in terms of pattern and intensity on the macrocycle concentration was also investigated. Supplementary Figure S2 (Supplementary Material) shows that the CD signal magnitude of (D)ZnP(-) films on glass slides increases at higher bulk solution concentrations without any perceptible changes in the chiral features. The calculated ratios between the maximum of the CD signals and recorded OD (see the *Experimental* section for details) shows that this parameter is substantially constant in the investigated range of concentrations (0.013, in the 0.5–1 × 10^−4^ M). Similar results have been obtained for the deposition of the (L) porphyrin enantiomer in the same conditions.

Comparing the spectral features of the (L)ZnP(-) film from toluene (Figure 1B, green trace) with the ones obtained for aggregates grown in EtOH/H_2_O (25:75 v/v) solutions (Figure 1B, blue trace), and one deposited at solid state (Figure 1B, red trace), a certain resemblance can be observed, even if the ones obtained for films casted by toluene show a higher degree of complexity, detectable with the emerging dichroic band at 449 nm.

**FIGURE 2:**
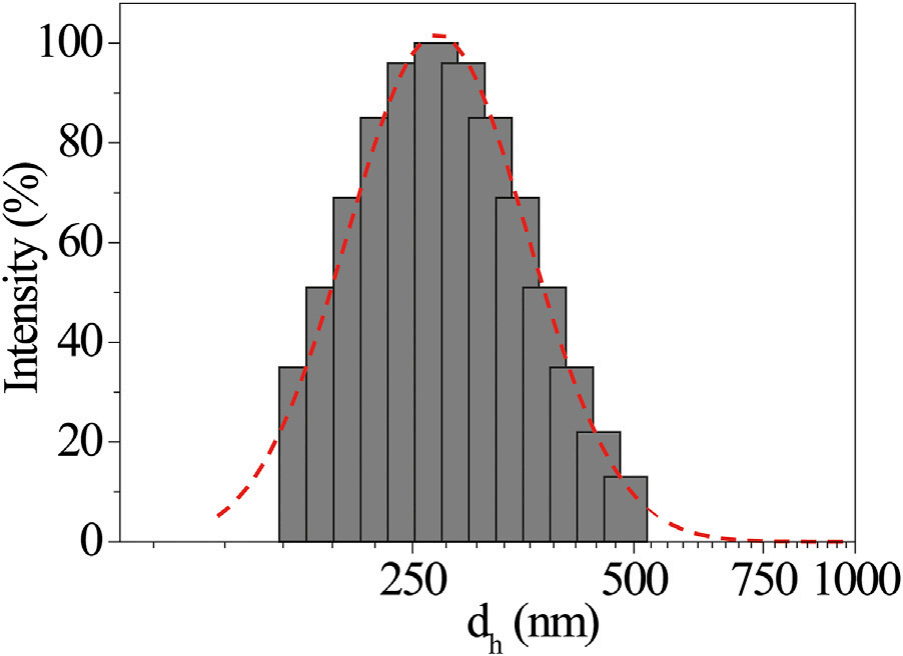
Representative dynamic light scattering (DLS) intensity size distribution of (L)ZnP(-) 10^−4^ M toluene solution, where the presence of self assemblies is detected. Gaussian fit in dotted line.

The close similarity between these systems allows us to invoke a like aggregation mode between porphyrin platforms during the film formation, involving the coordination of the proline carboxylate to the Zn^2+^ ion (Stefanelli et al., 2020b). It must be said that the aggregation conditions are rather different in the two cases. Indeed, in EtOH/H_2_O, the aggregation is boosted by hydrophobic effect, which can be surely excluded here in driving film formation. On the other hand, the chirality transfer during the self-assembly process on glass and the resulting nanostructured supramolecular chirality should be the result of several concomitant factors as, for example, the evaporation kinetic (Hattori et al., 2017). Moreover, it is abundantly reported that porphyrin aggregation often is based on the “sergeants-and-soldiers” principle, where a small set of specific aggregates acts as chiral seeds to catalyze the following aggregation process (Mammana et al., 2007). Based on these considerations, we decided to perform DLS studies to assess the eventual presence of protoaggregated species in the (L)ZnP(-) 10^−4^ M toluene bulk solutions (Table 1, entry 1). As shown in Figure 2, the sample presents aggregates with low polydispersity size distribution, centered at d_h_∼290 nm. Additionally, this distribution resulted to be influenced by porphyrin concentration. According to the comparative DLS analysis shown in Table 1, lowering the concentration down to 10^−5^ M is reflected in a lowering of the average size together with a significant increase in the size polydispersity (Table 1, entry 2). At 10^−6^ M, very small aggregates (∼50 nm size) were slightly detectable after high acquisition time (Table 1, entry 3). Experiment on long-term evolution of the size distribution would be desirable to understand whether such differences can be related to a change in the aggregation kinetics with porphyrin concentration. It is important to mention that toluene solutions of (D)ZnP(-) enantiomer were also checked under the same experimental conditions, showing no significant variation in the behavior of the size distribution, above reported for the (L)- counterpart.

**TABLE 1:** Dynamic light scattering (DLS) analysis of (L)ZnP(-) and the achiral ZnpCTPP in different solvents and concentrations.

Entry	Sample	Solvent	[M]	Aggregate	
				d_h_ [nm] ± σ	PDI
1	**(L)ZnP**	Toluene	10^–4^	290 ± 20	0.05
2	**(L)ZnP**	Toluene	10^–5^	160 ± 60	0.2
3	**(L)ZnP**	Toluene	10^–6^	50 ± 30	0.4
4	**(L)ZnP**	Ethanol	10^–4^	No aggregates	
5	**ZnpCTPP**	Toluene	10^–4^	No aggregates	

On the other hand, the involvement of Zn coordination on the chirality transfer from solutions to solid films is also suggested by the fact that films obtained from EtOH and THF are CD silent: In both cases, the remarkable coordination abilities toward Zn metal ions with respect to toluene make these solvents strong competitors for the aminoacidic residue. As a consequence, individual porphyrin macrocycles are well solvated, and the development of oligomeric structures is scarcely probable or driven to the formation of nucleation seeds below a critical threshold (Table 1, entry 4).

Additionally, ZnpCTPP is here utilized as a nonchiral probe since it also possesses a coordinated Zn(II) ion and a carboxylate group that should be able to drive the formation of ordered supramolecular assemblies. However, DLS studies disclose the absence of detectable aggregates in toluene at high concentration (Table 1, entry 5), proving the importance of the proline-appended group on the early aggregation stage. Furthermore, in case of films from nonchiral porphyrins, chiroptical signals should still emerge when symmetry-breaking effect of the surface, or stochastic formation of nucleation seeds with right- or left-handed orientation occur during the deposition (Chen et al., 2006). In this case, these events can be clearly ruled out by the fact that the CD signal of film from ZnpCTPP toluene solution on glass are silent.

To further investigate the network of interactions between solvent and porphyrins involved in both the aggregation process and in the effective chirality transfer to the solid state, we studied the drop-casted films of the corresponding free bases [(L)- and (D)H_2_P(-), Scheme 1] from THF and EtOH (toluene was excluded since these macrocycles are scarcely soluble in this solvent). Once again, films from THF did not feature any dichroic bands (data not shown). On the contrary, CD spectra of the spotted 10^−4^ M solutions of both enantiomers in EtOH displayed mirrored bisignated bands, although poorly reproducible in terms of intensity, sign, and spectral pattern depending on the batch solutions (data not shown). We have ascribed this evidence to the formation of small nucleation seeds of random overall symmetry, due to the lack of specific driving force such as the metal–ion coordination. The same behavior has been in fact observed upon aggregation of these species in hydroalcoholic solution (Stefanelli et al., 2020b) that results in the formation of supramolecular structures with very low g-factor.

DLS measurements support this hypothesis, since at the same concentration, (L)H_2_P(-) ethanol solutions showed a low tendency to form aggregates (which were rarely detectable at sizes in the range of 50–150 nm), suggesting a good solvation capacity of ethanol.

### Evaluation of the Influence of the Glass Substrate on Films

It is well known that several surface properties like wettability/nature and roughness are crucial for the film formation, affecting important mechanical as well as optical features, particularly at micro/nano scale. In our studies, we have investigated the influence of the glass surface on the chiroptical properties of the formed films by drop-casting toluene (L)/(D)ZnP(-) porphyrin solutions on glass slides of different source (i.e., commercial and of low roughness). A first difference among the films formed onto these two substrates was pointed out by the corresponding UV-vis spectra (Supplementary Figure S3), which showed a Soret band more red shifted and narrower for the layers casted on the ultraflat slide, indicative of a higher specificity in forming J-aggregated structures on such a substrate. Concurrently, CD measurements showed chiroptical features exclusively in the case of this film, pointing out a remarkable influence of the substrate roughness on the final porphyrin arrangement onto the surface in a chiral fashion.

**FIGURE 3:**
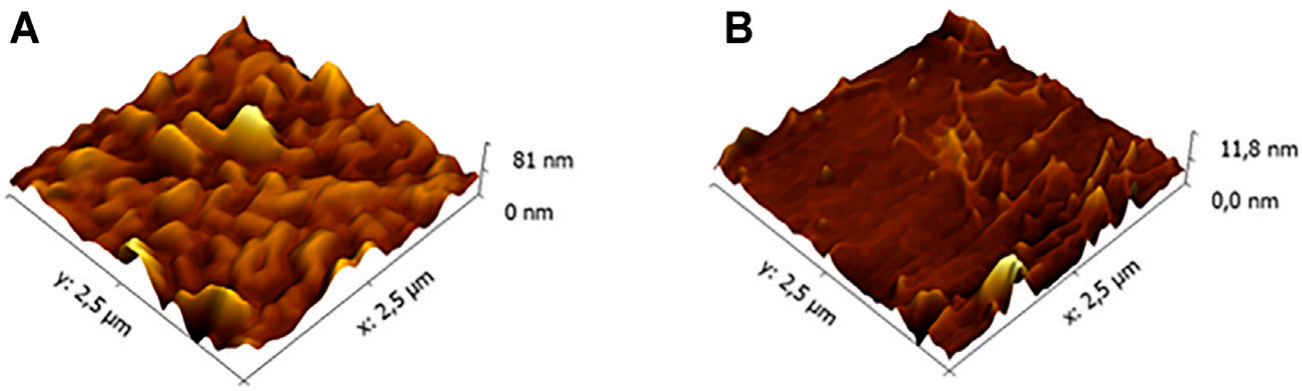
Atomic force microscopy (AFM) morphologies of **(A)** a commercial and **(B)** low-roughness glasses.

This interesting result led us to investigate more in depth the film morphology, and the results will be reported in a separate paper focused on this issue. Herein, we anticipate that the morphological investigation has highlighted a significant difference between the commercial glass and the low-roughness one. AFM images reported in the Figure 3 clearly show that the commercial glass (Figure 3A) is characterized by many hills and valleys with an overall height difference of about 75 nm. Conversely, the low-roughness glasses (Figure 3B) possess wide terraces, where the surface roughness is below 6 nm. Only in confined areas, we have observed some scratches that increase the overall roughness up to 10 nm. These evident differences between the two glass types can give a rationale of the observed porphyrin film chirality discussed above. Indeed, we can surmise that the porphyrin deposition onto a flat surface can facilitate the chiral organization of the macrocycles during the solvent evaporation. Conversely, the irregular commercial glass surface does not offer an optimal platform for a uniform film growth, which results in lack of stereospecificity.

Herein, we can conclude that the film chiral arrangement onto surface is due to a fine interplay of effects, i.e., the nature of the solvent, the concentration of the porphyrin monomer, and the nature of the deposition surface.

**FIGURE 4:**
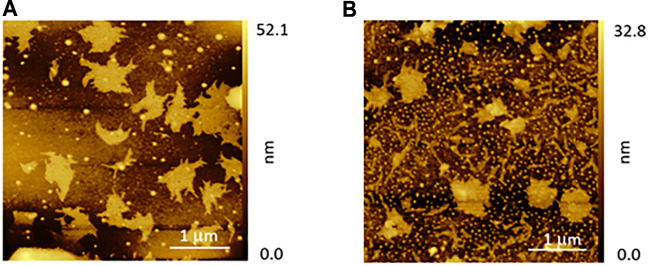
AFM morphologies of the (D)ZnP(-) film as a function of time: **(A)** as deposited and **(B)** after about 100 h with the sample taken in ambient conditions.

At the end of these investigations, we also evaluated some important film properties for applicative purposes, namely, the stability over time and temperature assessed by CD spectroscopy (Supplementary Figures S4A, B, respectively). We observed that the (L)/(D) porphyrin films casted by from toluene solution undergo a small rearrangement during 24–36 h and then remain stable until 72 h after the casting. At the end of this time, *ca.* 25% of the CD signal is lost and is kept constant for further 7 days. The organic layers are found more sensitive to heating, preserving their optical properties when exposed to temperature up to 50°C, and deteriorating for higher temperatures. In addition, AFM investigation gave a direct visualization of the film morphological evolution as a function of time. Figure 4 summarizes and compares the (D)ZnP(-) porphyrin film after about 100 h from the sample preparation. At the beginning, the morphology shows domains characterized by dendritic structures (Figure 4A), in good agreement with analogous systems reported in literature (Iavicoli et al., 2009). The film is not stable in ambient conditions, but undergoes a new assembling where the overall film roughness is slightly reduced (Figure 4B). It is reasonable that some solvent residuals, embedded inside the film after the deposition, are removed (e.g., evaporation) with a longer time interval, and a remarkable change in the porphyrin aggregates is produced.

### Conclusion and future perspectives

These studies report a convenient way to achieve chiral films by the efficient transfer of chirality from porphyrin molecular systems to film on glass by selecting a proper solvent for drop-casting process. The results obtained point out the crucial role played by nucleation seeds (small chiral porphyrin oligoaggregates), which are formed in solution. We found, in fact, that the use of oxygen donor solvents (EtOH and THF) well solvate the macrocycles either as free bases and zinc complexes, so hampering the effective supramolecular organization of the forming film in a chiral fashion. In analogy with our previous studies, the formation of chiral assemblies based on (L)/(D) prolinated porphyrins is guided by the coordination of the aminoacidic moiety to the zinc ion. For this reason, the use of toluene as solvent proves to be a good choice. Indeed, besides the noncoordinating character, toluene possesses also the highest surface tension (28.4, 26.4, and 22.1 mN/m for toluene, THF, and EtOH, respectively) and the lowest vapor pressure (29, 200, and 59 hPa at 20°C for toluene, THF, and EtOH, respectively), which favor the film adhesion to the substrate and prevent either the fast evaporation of solvent and excessive drying pattern formation, both essential conditions for a stereospecific growth of porphyrin aggregates during the self-assembly process. CD spectra evidence for films of both (L)- and (D)ZnP(-) have different patterns with respect to those observed in the case of the aggregation in solution. These results indicate the occurrence of specific interactions between the growing structures and the glass, whose roughness necessarily has to be around 1 nm for the effective chiral arrangement of macrocycles onto the surface. These findings would be of importance for the easy development of cost-effective molecular films to be potentially used in fields where chiral materials are highly desired, as asymmetric catalysis (Simonneaux et al., 2006; Mallat et al., 2007) and chiral sensors (Sannicolò et al., 2014; Shang et al., 2017; Stefanelli et al., 2019). In this regard, we can anticipate that the as-deposited films are fluorescent and highly stable when immersed in water solutions even for a prolonged period. Therefore, these features warrant for their use in optical chiral sensing measurements, which are currently ongoing in our laboratories and whose results will be reported in due course.

## Experimental Section

### General

Porphyrin derivatives investigated in these studies have been prepared as previously reported by our group (Stefanelli et al., 2020b) by using reagents and solvents of commercial sources (Sigma Aldrich, ChemImpex, TCI) in the highest degree of purity without further purifications. The casted films were analyzed by UV-vis spectroscopy using, according to the experimental needs, either a Varian Cary 100 or a JASCO J-1500, equipped with a thermostated cell holder set at 298 K, and purged with ultra-pure nitrogen gas. CD spectroscopic measurements were carried out by Jasco J-1500 equipped as just mentioned. The evaluation of the film optical density (OD) was performed consecutively by only changing the operative mode of J-1500 spectrophotometer without moving the sample, in order to irradiate the same spot of the porphyrin film in both absorbance and CD measurements. Different traces at different stock concentrations were taken, referenced against untreated blank glass slide. Linear dichroism contribution (LD) has been found to be <0.0004 DOD units in all the cases examined. Also, to rule out the strong contributions of birefringence effects, both front and back of the slide were measured without evidencing significative changes in the CD profiles.

### Porphyrin Film Preparation

The porphyrin solutions to be casted on glass slides were prepared as follows: 10^−4^ M solutions of macrocycles in 4 ml of the chosen solvent (ethanol, tetrahydrofuran, or toluene) were kept under sonication for 2 min (Fisher Scientific FB15047 apparatus). The solution was then filtered first through a 0.45-μm and then a 0.22-μm FilterBio ${\;}^{®}$ syringe filters, sonicated again, and left to stand for 1 h before casting. Concentrations were confirmed by UV-vis spectroscopy. The porphyrin solution should be used within 2 weeks from preparation, to ensure optimal and reproducible results.

Microscope glass slides (SuperFrost ${}^{®}$ , Menzel) cut into 76 mm  ×  26 mm and ultra-flat quartz-coated glass substrate (Ossila, 25 mm × 25 mm) pieces were used for deposition as received. Porphyrin solution (10 μl) was layered on the glass slide surface by drop-casting method. The drop was left to dry at standard laboratory conditions, until complete solvent evaporation. The time of evaporation and dimension of spots strictly depend on the solvent used. Glass pieces can be reused after removing the organic layers by extensive washing with a suitable organic solvent (usually chloroform or dichloromethane). Afterward, the slide is treated with acetone and gently dried utilizing a flow of pure nitrogen.

### Dynamic Light Scattering Studies

Size distribution by dynamic light scattering (DLS) was performed with Hamamatsu HC120 photometer (Brookhaven, NY, United States), equipped with a BI-200SM goniometer, a BI-9000 AT photocorrelator allowing to detect a particle size dynamic range from 5 to 5,000 nm, a solid-state laser (Suwtech Inc., SHA, CN) with a wavelength of 532 nm powered by a power supply LDC-2500 (Suwtech Inc.), and a phototube (Hamamatsu Photonics K.K., JP). The solvents used for the sample dissolution were prefiltered with a 0.22-μm hydrophobic filter (FilterBio ${\;}^{®}$ ). Eight hours after the sample preparation, a volume of 500 μl of stock solution was transferred into a quartz cuvette and maintained at a temperature of 25° C using an external F30-C thermostat (Julabo GmbH, DE), circulating water in a coil placed in the vat containing the refractive index matching liquid. Acquisition time was set to 6 min. The size of chiral porphyrin assemblies was estimated by the hydrodynamic diameter (d_h_) distributions according to the Stokes–Einstein equation. The autocorrelation functions were analyzed using the CONTIN algorithm of the Dynamic Light Scattering Software ver. 3.18 provided by Brookhaven Instruments. A Gaussian fitting procedure was applied to the intensity distribution of d_h_ using the OriginPro 8.1 software for extrapolating average d_h_ values ± standard deviation from three independent measurements. Polydispersity index (PDI) was calculated from cumulant analysis of the measured intensity autocorrelation function.

### Atomic Force Microscopy Measurements

A commercial Keysight 5500 atomic force microscopy (AFM) equipment was employed for the sample topographic analysis. Images were collected in noncontact mode to prevent possible damages of the porphyrin aggregates during contact measures. Silicon NanoSensors tip were used with a resonance frequency in air of about 300 kHz. The apex radius is about 20 nm. After the acquisition, images were analyzed by the free Gwyddion software.

## Data Availability

The original contributions presented in the study are included in the article/Supplementary Material. Further inquiries can be directed to the corresponding author.
