# Supplementary material for: Seeding Chiral Ensembles of Prolinated Porphyrin Derivatives on Glass Surface: Simple and Rapid Access to Chiral Porphyrin Films
Source: Front Chem. 2022 Jan 31;9:804893. doi: 10.3389/fchem.2021.804893 (PMC8841355; doi:10.3389/fchem.2021.804893)
Supplement: Supplementary file 1 [file DataSheet1.docx]

Supplementary Material


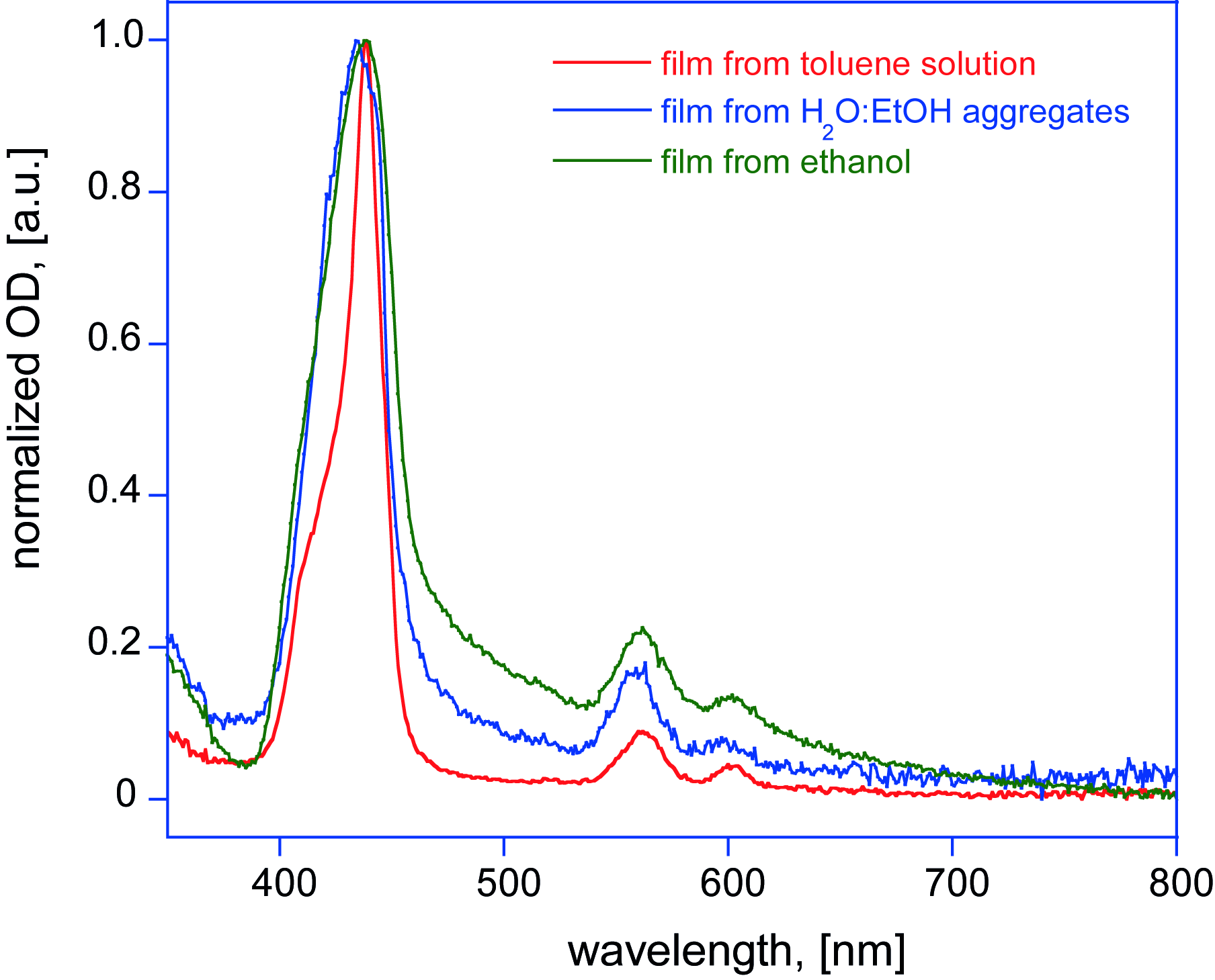


**Supplementary Figure S1.** UV-Vis spectra of **(L)ZnP(-)** casted films from different solutions.


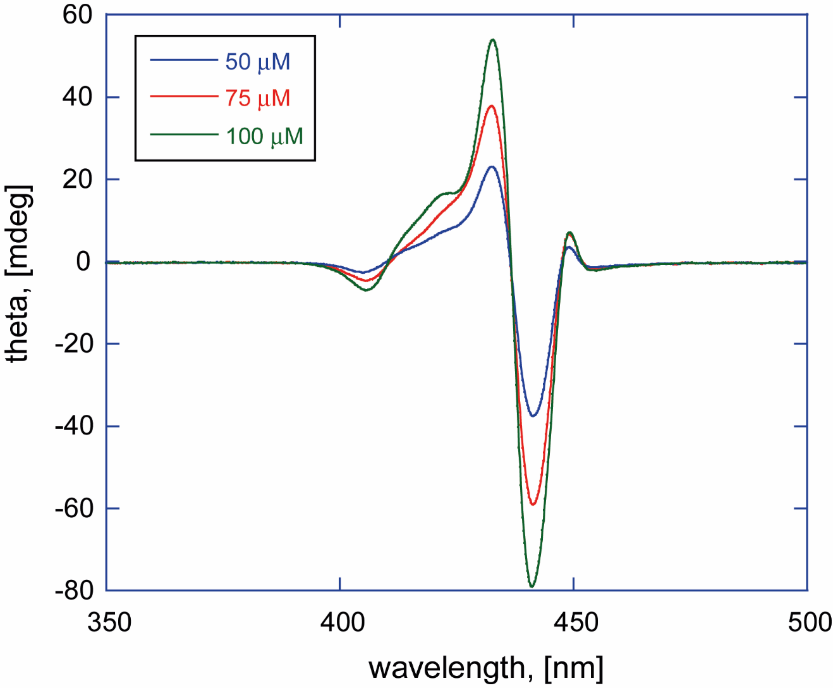


**Supplementary Figure S2**: **(D)ZnP(-)** films on glass from toluene solutions at different concentrations.


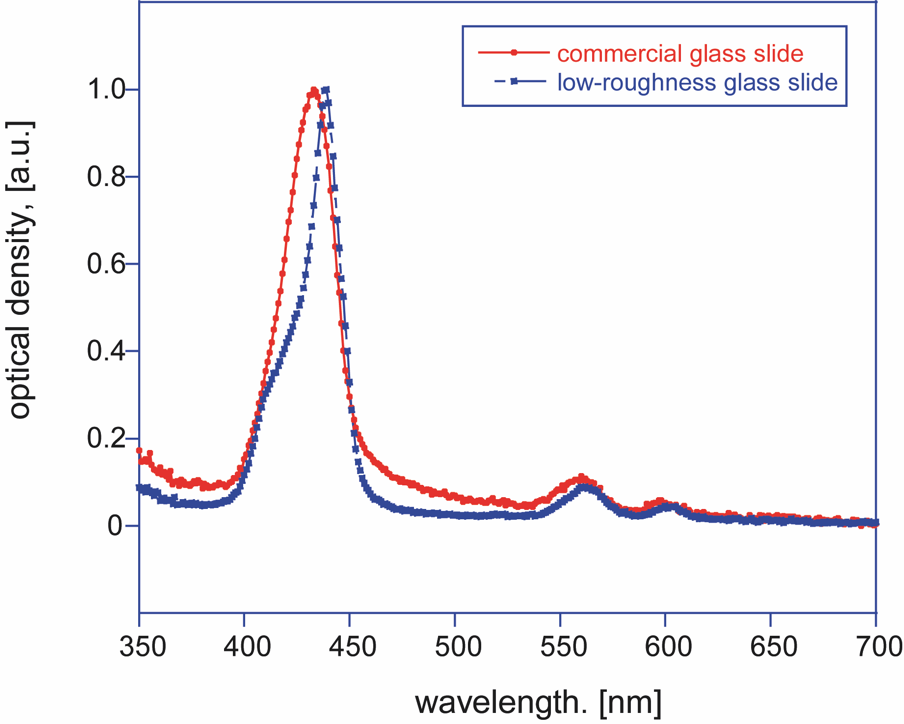


**Supplementary Figure S3**: UV-Vis spectra of **(L)ZnP(-)** casted film from 10^-4^ M toluene solution onto non-flat glass slide (red trace) and onto ultraflat glass slide (blue trace).


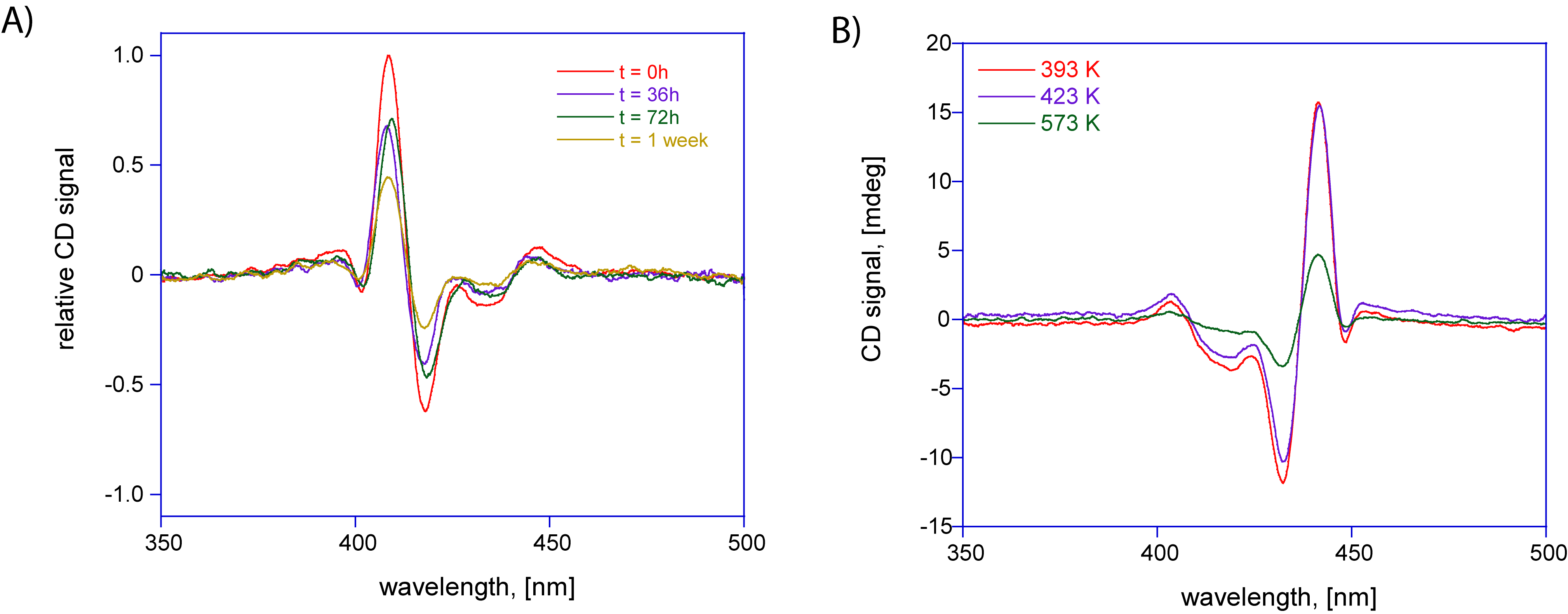


**Supplementary Figure S4.** Stability over time (A) and with increasing temperatures (B) of the **(L)ZnP(-)** casted film from 10^-4^ M toluene solution onto glass.
